# Conjunctival pyogenic granuloma following insect bite

**Published:** 2022-06-07

**Authors:** Suchetha Ireni, Sanjay Bhargav, Kavya Madhuri Bejjanki, Padmaja Kumari Rani

**Affiliations:** 1 Uveitis Fellow: LV Prasad Eye institute, Hyderabad, India.; 2 Vision Technician: LV Prasad Eye institute, Ashwapuram, India.; 3 Assistant Ophthalmologist: Ophthalmic Plastic Surgery and Ocular Oncology Services, LV Prasad Eye institute, Vijayawada, India.; 4 Network Associate Director, Teleophthalmology: LV Prasad Eye Institute, Hyderabad, India.

A woman in her thirties came to our primary eye care centre (vision centre) complaining of foreign body sensation and discharge in her right eye which she had been experiencing for three weeks. She had been bitten in the eye by an ant and an insect particle had been removed from the eye one week before the onset of symptoms. She had applied topical antibiotics that were prescribed locally but there was no improvement.

**Figure 1:**
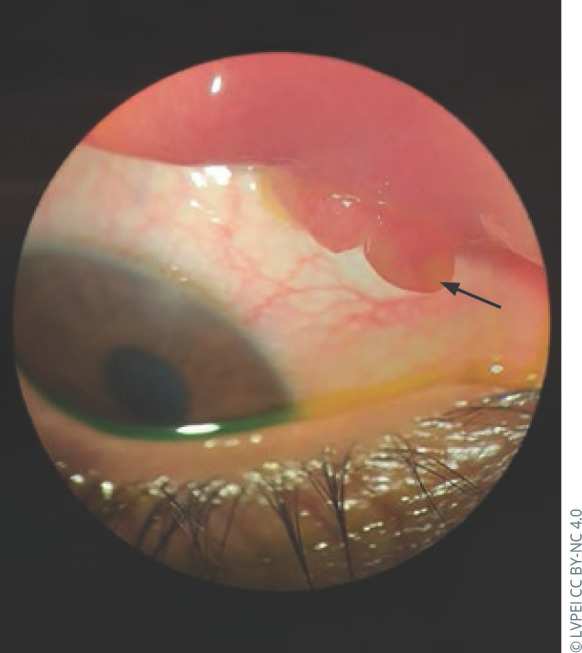
Pedunculated vascular fleshy growth (arrow) on palpebral conjunctiva of upper eyelid (right eye).

On examination at the vision centre, the vision in the right eye was 20/20. All the anterior segment findings were normal. However, upon eversion of the eyelid, a reddish-pink vascular pedunculated lesion was visible on the palpebral conjunctiva of the upper eyelid of the right eye (Figure 1).

The vision technician took a photograph (Figure 1) using the camera on a smart tablet and uploaded the photograph to the patient's cloud-based electronic medical record. The medical record could be seen by the ophthalmologists based at the central hospital's teleophthalmology centre. Teleconsultation with the ophthalmologist was requested and a provisional diagnosis of pyogenic granuloma was made. The patient was promptly referred to the central hospital (a tertiary centre) for further management.

**Question 1**

**What is pyogenic granuloma?**

- A vascular lesion of infectious aetiology
- A benign, non-tender, exuberant proliferation of granulomatous tissue in response to trauma or surgery
- A malignant vascular proliferative lesion
- A tender granulomatous lesion of inflammatory aetiology

**Question 2**

**What is the key differential diagnosis for conjunctival pyogenic granuloma?**

- Dermolipoma
- Dermoid cyst
- Conjunctival concretions
- Squamous cell carcinoma

**Question 3**

**What are the management options for conjunctival pyogenic granuloma?**

- Topical antibiotics, hot fomentation, lid hygiene
- Topical corticosteroids, removal of inciting agent, excision biopsy, pulsed dye laser
- Topical lubricants and lid scrubs with baby shampoo
- Topical anti-inflammatory agents and oral analgesics

**ANSWERS**

1. **b.** Pyogenic granuloma is a benign, exuberant proliferation of granulomatous lesions due to angiogenic imbalance as a result of of minor trauma, foreign body, or surgery. Eversion of the eyelid is important in order to correctly diagnose these lesions.
2. **d.** The term pyogenic granuloma is a misnomer, as the lesion is neither infectious nor inflammatory in nature. It is a benign, non-tender lesion. Differentiating from malignant lesions is important in order to avoid unnecessary treatment. The main difference is the slow growth of malignant lesions in contrast to the rapid growth of pyogenic granulomas.
3. **b.** Conjunctival pyogenic granulomas of smaller size usually respond to topical corticosteroids or pulsed dye lasers. Pulsed dye lasers are effective and safe for the treatment of small and solitary pyogenic granulomas, treated with a vascular-specific (585 nm), pulsed (450 microseconds) dye laser using a 5 mm spot size with a laser energy of 6 to 7 J/cm^2^. Many patients will eventually require surgical excision of the granuloma along with the removal of the inciting agent or irritant. Excision biopsy allows histological confirmation, and is helpful if there is any doubt about the diagnosis.

